# Electronic Response and Charge Inversion at Polarized Gold Electrode

**DOI:** 10.1002/anie.202413614

**Published:** 2024-11-04

**Authors:** Linnéa Andersson, Michiel Sprik, Jürg Hutter, Chao Zhang

**Affiliations:** ^1^ Department of Chemistry-Ångström Laboratory Uppsala University Lägerhyddsvägen 1, BOX 538 75121 Uppsala; ^2^ Department of Chemistry University of Cambridge Lensfield Rd Cambridge CB2 1EW United Kingdom; ^3^ Institut für Chemie Universität Zürich Winterthurerstrasse 190 CH-8057 Zürich Switzerland

**Keywords:** Double layer, Molecular dynamics, Electronic properties, Electrified interface, Anion adsorption

## Abstract

We have studied polarized Au(100) and Au(111) electrodes immersed in electrolyte solution by implementing finite‐field methods in density functional theory‐based molecular dynamics simulations. This allows us to directly compute the Helmholtz capacitance of electric double layer by including both electronic and ionic degrees of freedom, and the results turn out to be in excellent agreement with experiments. It is found that the electronic response of Au electrode makes a crucial contribution to the high Helmholtz capacitance and the instantaneous adsorption of Cl can lead to a charge inversion on the anodic polarized Au(100) surface. These findings point out ways to improve popular semi‐classical models for simulating electrified solid–liquid interfaces and to identify the nature of surface charges therein which are difficult to access in experiments.

## Graphical abstract

A new first‐principles‐based simulation method to model the electrified metal‐electrolyte interface has been developed and applied to both Au(100) and Au(111) surfaces in NaCl solution. It reveals that the electronic response of Au electrode contributes significantly to the experimental reported high capacitance and the unexpected instantaneous adsorption of Cl ions at anodic polarized Au (100) (instead at PZC) can lead to a charge inversion.

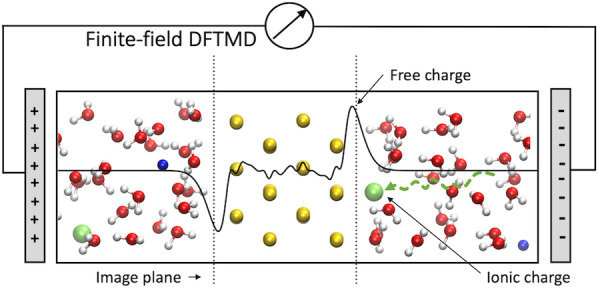

## Introduction

The metal‐electrolyte interface is prototypical in electrochemistry and highly relevant for energy storage, conversion and corrosion processes. In the boundary region between metal electrode and electrolyte solution, an electric double layer (EDL) is formed when the excess electronic charge on the metal surface is compensated by equal and opposite ionic charge in electrolyte solution. This happens when the system is outside of so‐called the potential of zero charge (PZC).^[1]^ Despite of decades of efforts,[^2^, ^3^] EDLs at the metal‐electrolyte interfaces are still full of surprises. This is evidenced by recent experimental reports of a strong deviation from the Gouy‐Chapman theory for the diffuse double layer,^[4]^ an unexpected high capacitance of the metal nanoparticle‐water interfaces,^[5]^ and different ionic responses from Na^+^ and Cl^−^ shown in the absorption THz spectra.^[6]^

Understanding the molecular structure of EDLs at the metal‐electrolyte interface requires inputs from theoretical investigations. Molecular modelling built on the principle of quantum mechanics and statistical mechanics^[7]^ are most suitable to unveil the factors that contribute to the double‐layer structures and their effects on electrochemical activity. Significant progress in this area has been made recently, which points out the importance of water dynamics,^[8]^ the chemisorption of water,[^9^, ^10^] and specific ion adsorptions.^[11]^ Nevertheless, a direct computation of the Helmholtz capacitance of electrified metal‐electrolyte interfaces which is a key experimental observable is not fully available from first‐principles‐based molecular dynamics simulations.

This gap between experimental and theoretical investigation of EDLs at metal‐electrolyte interfaces is partly due to the challenges of molecular modelling of electrified interfaces. There are in general three different categories of approaches to model electrified interfaces within density functional theory (DFT) or density functional theory‐based MD (DFTMD) simulations:^[12]^ grand‐canonical,[^13^, ^14^, ^15^, ^16^, ^17^, ^18^, ^19^, ^20^] counter‐ion[^10^, ^21^, ^22^, ^23^, ^24^]/pseudo‐atom^[25]^ and finite‐field. Grand‐canonical approaches are usually parameterized with implicit solvation models[^26^, ^27^] where the Helmholtz capacitance (commonly quoted as 20 *μ*F/cm^2^) is an input quantity rather than a prediction. In the counter‐ion/pseudo‐atom approaches, ions in electrolyte solution are either out of their equilibrium positions or simply neglected. This renders the difficulty of being certain about the computed capacitance from DFTMD simulations with these methods and make it less useful to guide semi‐classical,[^28^, ^29^] quantum‐mechanical/molecular mechanical (QM/MM)^[30]^ and hybrid solvation^[31]^ types of approaches. On the other hand, finite‐field methods, in particular with constant electric displacement *D* Hamiltonian,^[32]^ have been shown to be rather useful in investigating the dielectric response of polar fluids,[^33^, ^34^] bulk electrolyte solutions,^[35]^ polar surfaces,[^36^, ^37^] and the protonic double‐layer at insulating metal‐oxide/electrolyte interfaces.[^38^, ^39^] Despite of that, its implementation for electrified metallic electrodes immersed in electrolyte solution beyond a semi‐classical approach[^40^, ^41^, ^42^] is long awaited.

**Figure 1:**
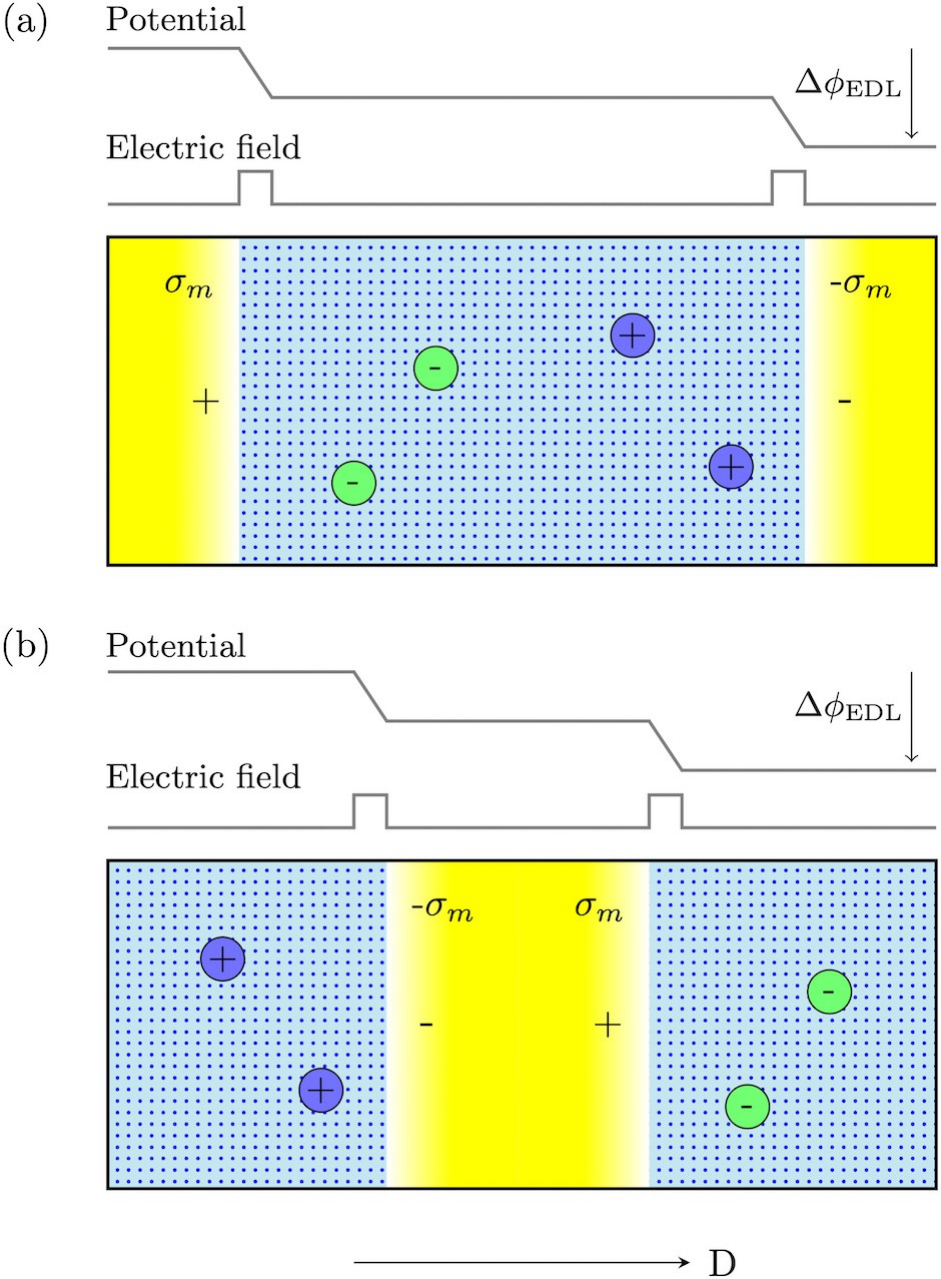
(a) and (b) Two equivalent supercell representations of electrified interfaces between metal electrode and electrolyte solution under finite electric displacement *D* and periodic boundary conditions. $\sigma_{m}$ is the free charge density at metal surface and $\Delta \varphi_{\mathrm{EDL}}$ is the sum of the potential over two double layers. Electric‐field profile (not the corresponding potential profile) is translational invariant, which ensures a continuous and smooth dynamics.

In the finite‐field treatment of electrified interfaces under periodic boundary conditions, there is only one metal slab, which is polarized by the *D* field (see Figure 1). The metal slab as a whole remains neutral and so does the electrolyte slab. Since the metal slab is an electronic conductor while the electrolyte slab is an electronic insulator, the (electronic) surface charge density on the metal $\sigma_{m}$ is related to the applied *D* field by $D=4\pi \sigma_{m}$ . The resulting potential across the supercell of length *L* is $\Delta \varphi =-\left(D-4\pi P\right)L$ , as guaranteed by the modern theory of polarization^[43]^ *P* and the Stengel‐Spaldin‐Vanderbilt constant *D* Hamiltonian.^[32]^ For electrified interfaces, this (cell) potential Δφ equals to the sum of the potential over two double layers $\Delta \varphi_{\mathrm{EDL}}$ because the electrolyte is an ionic conductor and the Maxwell electric field over the electrolyte vanishes (on statistical average).

In this work, we have implemented DFTMD simulations for modelling electrified interfaces of Au/NaCl (aq) under finite electric displacement *D*, which allows us to directly compute the Helmholtz capacitance for both Au(100) and Au(111) surfaces with ionic and electronic degrees of freedom treated on an equal footing. The Helmholtz capacitance that we obtained is about 60 *μ*F/cm^2^, in excellent agreement with (recent) measurements for single crystal Au electrodes with non‐specific ion adsorption. The high value of this capacitance we found in our simulations can be understood by studying the electronic response of the Au electrodes and verified by tuning the position of image plane in the semi‐classical models for exactly the same chemical composition of the system and electric boundary condition. Unexpectedly, we observed fast Cl adsorption on both Au(111) at PZC and Au(100) with positive free charge within the time scale of DFTMD simulations. This leads to a charge inversion on the anodic polarized Au(100) which produces almost identical water orientational distribution as that found on cathodic polarized Au(100) without Cl adsorption. These observations suggest a non‐monotonic charging behavior of Au(100) with positive bias.

## Results and Discussion

### DFTMD Simulations of Au/NaCl(aq) Under Finite *D*

**Figure 2:**
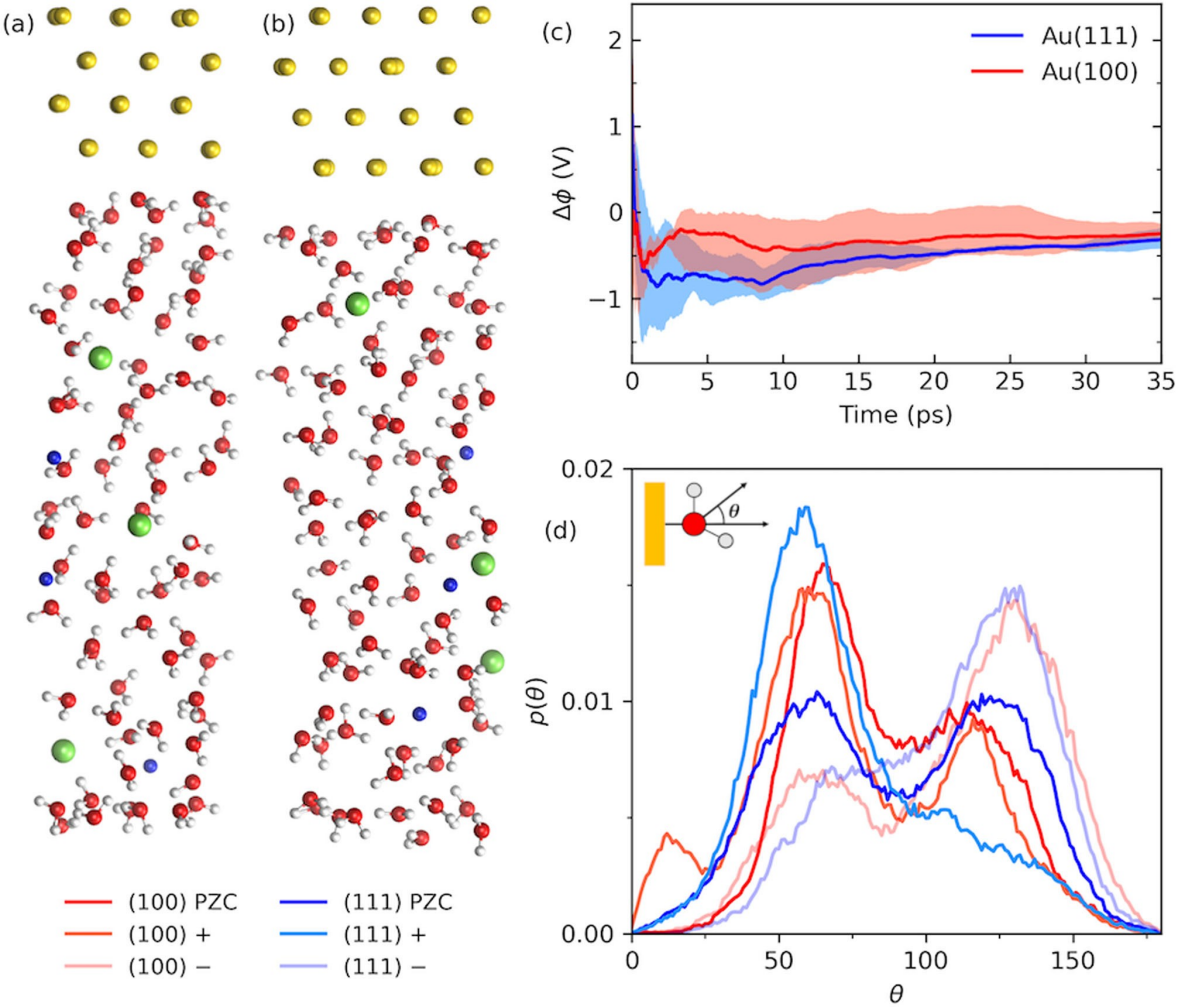
(a)‐(b) Snapshots from the finite‐field DFTMD trajectories at *D*=0.018 au ($\sigma_{m}$ =8.2 *μ*C/cm^2^) for Au(100)/NaCl(sol) and Au(111)/NaCl(sol) respectively. Au: yellow; O: red; H: white; Na: blue; Cl: green. The salt concentration is about 2 molality (see the Supporting Information for computational setups). (c) Cumulative average of the cell potential Δφ for both the Au(100) and Au(111) systems. Each line was the average over 3 independent trajectories. (d) The distribution of the angle between the bisector of water with the surface normal for both Au(100) and Au(111) at PZC and *D*=0.018 au for the water within 4 Å of the surface. “+“ indicates anodic polarization and “−” indicates cathodic polarization.

To begin with, both Au(100)/NaCl(aq) and Au(111)/NaCl(aq) system (Figures 2a and b) were well equilibrated with semi‐classical MD simulations under the same electric boundary conditions before porting the system into DFTMD simulations (See Supporting Information for details). This has been shown to be very useful to speed up the convergence of capacitance calculations.[^38^, ^44^]

The Helmholtz capacitance can be computed using the formula shown below (Eq. 1), similar to the one used in the case of insulting oxide with proton charge.[^38^, ^45^] However, a key distinction here is that DFTMD simulations have been carried out under finite *D* and the imposed surface charge ($\sigma_{m}=D/4\pi$ ) is electronic (free) charge on the metal surface instead of adsorbed proton charge due to the acid‐base chemistry at different pH values.^[46]^

(1) $$C_{H}=2\left(\frac{d\sigma_{m}}{d\Delta \varphi }\right)=\frac{D}{2\pi \left(D-4\pi \left\langle P_{z}\right\rangle \right)L_{z}}$$

where Δφ is the sum of the potential differences at two sides of the same metal electrode (therefore a factor 2 for the averaged Helmholtz capacitance), *P_z_* is the Berry phase polarization^[47]^ (see Supporting Information for further explanation) and *L_z_* is the length of simulation box in the direction perpendicular to the interface.

Figure 2c shows the cumulative average of the cell potential Δφ (Eq. 1) over 35 ps of DFTMD under applied field D=0.018 au ($\sigma_{m}$ =8.2 *μ*C/cm^2^). They are averages of 3 independent trajectories for Au(100)/NaCl(aq) as well as for Au(111)/NaCl(aq) (see Figure S11 in the Supporting Information for the cumulative average of each individual trajectory). The resulting capacitance value for Au(100) is higher (66 *μ*F/cm^2^) than the value for Au(111) (52 *μ*F/cm^2^), however, the standard deviations of the potential are overlapping in these two cases which makes them indistinguishable in practice. These trajectories shown in Figure 2 are free from any specific ion adsorption (see Figure S9 in the Supporting Information for the distance of the nearest counter‐ions to the polarized surface). To put these numbers into perspective, the Helmholtz capacitance of the Au(100) single crystal in the absence of specific anion adsorption and at comparable surface charge density is about 65 *μ*F/cm^2^ from impedance spectroscopy^[48]^ and that of the Au(111) single crystal is about 72 *μ*F/cm^2^ from cyclic voltammetry with the Parsons‐Zobel analysis.^[4]^ Therefore, the agreement between theoretical and experimental values is excellent for Au electrodes without specific ion adsorption by considering the uncertainties involved both simulations and measurements.

The orientation of surface water molecules is known to shift in response to the surface charge of metal electrodes. Although the vibrational density of states of water bilayer look quite similar between Au(111) and Au(100),[^49^, ^50^] the response in a single water molecule to electric field was weaker for the surface with higher Au−O adsorption energy.[^51^, ^52^] Nevertheless, how surface water molecules reorient themselves at electrified Au surfaces immersed in electrolyte solutions is less clear and conclusive.[^53^, ^54^]

In Figure 2d the distribution of the angle between water bisector and the surface normal is plotted for gold electrodes at the PZC as well as for the negatively and positively charged surfaces. The corresponding oxygen and hydrogen density distributions are shown in Figures S5–S6 in the Supporting Information. At the PZC there are two peaks centered at around 60 and 120 degrees for both crystal faces. At these angles one of the O−H bonds is close to parallel with the surface while the other is pointed towards the bulk solution at 60 degrees and towards the surface at 120 degrees. The peaks therefore indicate an alternating H‐up/H‐down configuration typically present at (111) surfaces[^53^, ^55^] as a result of hydrogen bonding. For Au(100) the H‐up orientation is preferred which is in accord with the hydrogen distribution at PZC (Figure S5 in the Supporting Information). For the positively charged surface, we expect to see a shift toward smaller angles as the oxygen turns toward the surface. Such a shift was indeed observed for both Au(100) and Au(111) surfaces in our finite‐field DFTMD simulations. There is a single peak at 60 degrees for Au(111) and the appearance of a third peak at 20 degrees for Au(100), while the peak at 120 disappears for Au(111) and is diminished for Au(100). At the negatively charged surfaces we see a smaller peak at 60 degrees and a larger peak at 130 degrees. This shift comes from hydrogen turning toward the surface, as confirmed by the hydrogen distribution at D=0.018 au (see Figures S5–S6 in the Supporting Information). Therefore, our results confirm previous studies of water orientation at PZC for Au(111)^[53]^ but show a clear shift in the peak positions for electrified surfaces in addition to the pronounced intensity.^[54]^ Moreover, we find that water at Au(100) is more responsive to the negative bias while water at Au(111) is more responsive to the positive bias.

### Electronic Response of Au Electrodes

The high capacitance seen at metal/water interfaces has to do with the contribution from the electronic response of the metal electrode. The origin of this negative contribution was attributed either to the response of electronic spillover using the jellium model for the sp metals[^56^, ^57^, ^58^] or to the response of contact potential between adsorbed water molecules and metal electrode by Cheng and co‐workers using DFTMD and counter‐ion methods.[^59^, ^60^] However, what the response of electronic spillover looks like for a realistic d‐block metal using modern electronic structure calculations and how that changes upon the contact with electrolyte solution is not known. These are precisely the types of questions that finite‐field methods can answer. In particular, we can leverage on the freedom to switch between the full DFT Hamiltonian implemented in the CP2K code[^61^, ^62^] and the semi‐classical Hamiltonian implemented in the MetalWalls code[^63^, ^64^] for the same chemical composition of the system under the same electric boundary conditions.

**Figure 3:**
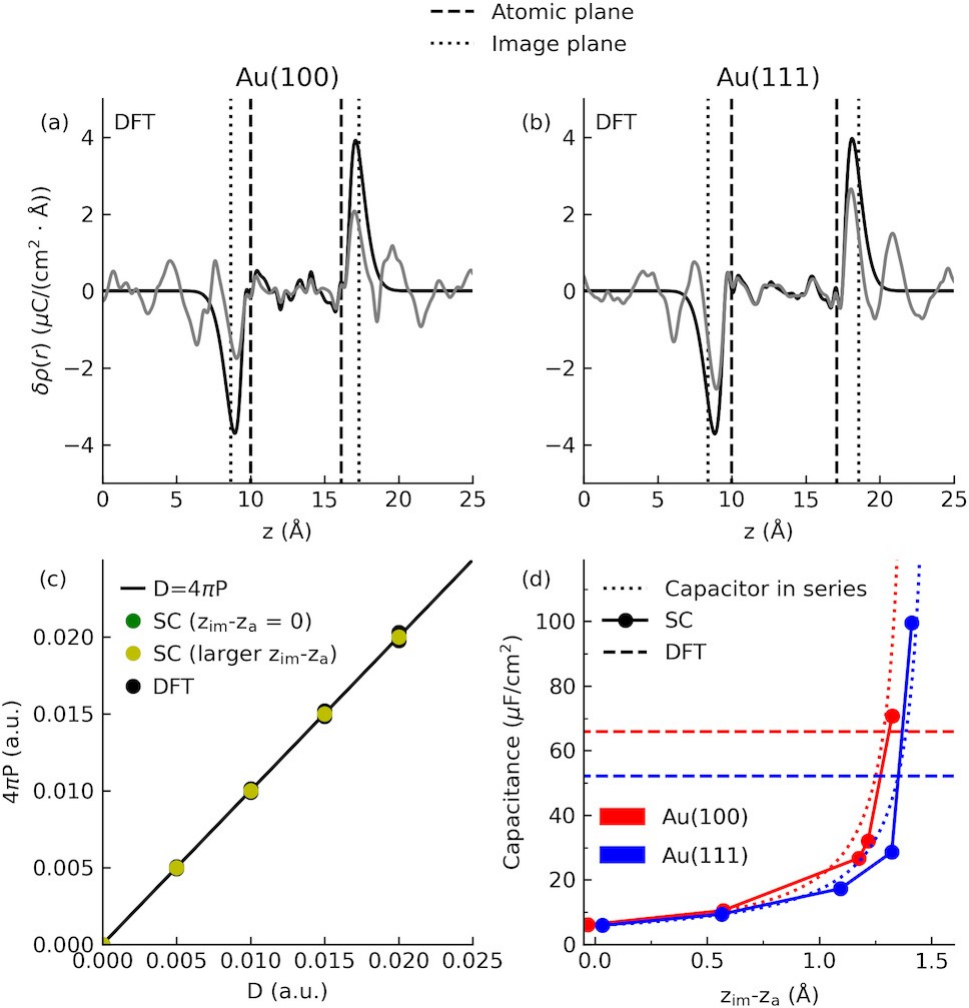
(a)‐(b) Charge density response from DFT for the Au(100) and Au(111) slabs at D=0.018 au. In vacuum (with the image plane shown): black; in electrolyte: grey. (c) Relationship between the D‐field and the metal polarization (Eq. 3) for both Au(100) and Au(111) with DFT and the semi‐classical (SC) model. (d) The double‐layer capacitance at the gold‐electrolyte interface as a function of image plane *z*_im_‐*z_a_* (Eq. 2) from finite‐field simulations with SC models. The theoretical lines from the capacitor‐in‐series model (see Eq. 5 and texts around for details) and the reference values obtained from finite‐field DFTMD simulations are also shown.

Figures 3a and b show how Au(100) and Au(111) respond to a finite *D* field in vacuum and in contact with electrolyte solution. It can be seen that the response of electronic charge to the finite *D* on each side of electrode is asymmetric in both vacuum and in contact with the electrolyte solution. This is also manifested by the difference between the atomic plane *z*_a_ and the image plane *z*_im_. The image plane calculated from the response charge density has a direct connection to the metal capacitance as it reflects the change in surface potential ∂χ with respect to surface charge density $\partial \sigma_{m}$ as:^[65]^

(2) $$z_{\mathrm{im}}-z_{a}=\frac{\int z\delta \rho \left(z\right){|}_{D}dz}{\int \delta \rho \left(z\right){|}_{D}dz}-z_{a}=-\epsilon_{0}\frac{\partial \chi }{\partial \sigma_{m}}=-\frac{1}{C_{M}}.$$

where *δ* $\rho \left(z\right){|}_{D}=\rho_{D}\left(z\right)-\rho_{D=0}\left(z\right)$ is the response charge density. It is found that the image plane position *z*_im_ (relative to the atomic plane *z_a_*) is 1.22 Å for Au(100) and 1.49 Å for Au(111). Comparing the charge response of Au electrode in vacuum and in solution, it is clear that charge transfer between adsorbed water and Au electrode does the modify response function and therefore the profile. However, the peak position in the charge response remains almost the same.

Before discussing the impact of the image plane on the total capacitance, one may wonder how to validate the physical significance of *z*_im_ computed using Eq. 2. Lang and Kohn showed that the definition of image plane according to Eq. 2 recovers the classical expression for the image potential.^[65]^ Here we looked into this question by considering how dielectric response works for metal. A metal should completely screen the electric field. This means the following relation must hold true:

(3) $$D=4\pi P_{M}=\frac{4\pi M_{z}}{A\cdot L_{M}}$$

where *P*_M_ is the polarization of the metal, *M_z_* is the dipole moment, *A* is the slab area and *L*_M_ is the length of the metal slab.

We found that in order to recover this relation between *D* and *P*_M_ in Eq. 3, *L*_M_ needs be the distance between the image planes,

(4) $$L_{M}=z_{\mathrm{im}}^{R}-z_{\mathrm{im}}^{L}.$$

This has been born out for both DFT and semi‐classical Hamiltonians (with very different positions of image plane), as shown in Figure 3c.

Then, the question is what will be the effect of *z*_im_ on the total capacitance. As mentioned before, when the metal is in contact with solution, the total capacitance can be considered as a capacitor‐in‐series,^[56]^

(5) $$\frac{1}{C_{\mathrm{tot}}}=\frac{1}{C_{\mathrm{sol}}}+\frac{1}{C_{M}}$$

where *C*_sol_ is the solution capacitance and *C*_M_ is the metal capacitance. We assume *C*_sol_ as the capacitance with $z_{\mathrm{im}}-z_{a}$ =0 from the semi‐classical simulations, i.e. 5.95 and 6.09 *μ*F/cm^2^ for Au(111) and Au(100) respectively. These baseline values are comparable with those obtained in previous studies with similar types of models.[^29^, ^66^] Therefore, Eq. 5 provides a theoretical prediction on the effect of image plane on the total capacitance, which can be checked with simulations.

As shown in Figure 3d, we do see a significant increment in the total capacitance when increasing the position of image plane in the semi‐classical simulations. The relationship between the image plane and the Gaussian width parameter in semi‐classical models for both Au(100) and Au(111) can be found in Figure S2 and S3 in the Supporting Information. Results from these simulations agree quite well with those predicted using Eq. 5. Interestingly, when the total capacitance from the semi‐classical simulations for Au(100) reaches the value of capacitance computed from finite‐field DFTMD simulations, the corresponding image plane $z_{\mathrm{im}}-z_{a}$ of 1.29 Å is close to that we found for Au(100) slab in vacuum with DFT ($z_{\mathrm{im}}-z_{a}$ =1.22 Å). However, the corresponding value for Au(111) obtained from DFT appears to be too large and go beyond the singularity point (−$C_{M}=C_{\mathrm{sol}}$ ). This is likely to do with the overestimation of the work function for our Au(111) slab (see Table S2 in the Supporting Information). Instead, taking the cross‐over point between the theoretical line using Eq. 5 and the capacitance value obtained from the finite‐field DFTMD simulations, one gets $z_{\mathrm{im}}-z_{a}$ for Au(111) is about 1.35 Å instead. This value is almost the same as the assumed position of image plane for Au(111).^[67]^

In addition, we have also seen the adsorption of Na ions in the semi‐classical simulations with $z_{\mathrm{im}}-z_{a}$ =1.22 Å (Figure S9 in the Supporting Information), similar to what was reported previously.^[68]^ This suggests a reparameterization of force fields for Na ions or the introducing of explicit polarizability is probably needed to restore the correct molecular structure at the electrified interface in semi‐classical MD simulations. Nevertheless, it is clear now that the origin of this enhancement in the total capacitance have little to do with the adsorption of Na ions but come from the electronic response of gold electrode. Therefore, what is shown here provides a stepping stone for understanding the unexpected high capacitance of single Au nanoparticle reported experimentally.^[5]^

### Adsorption of Cl

**Figure 4:**
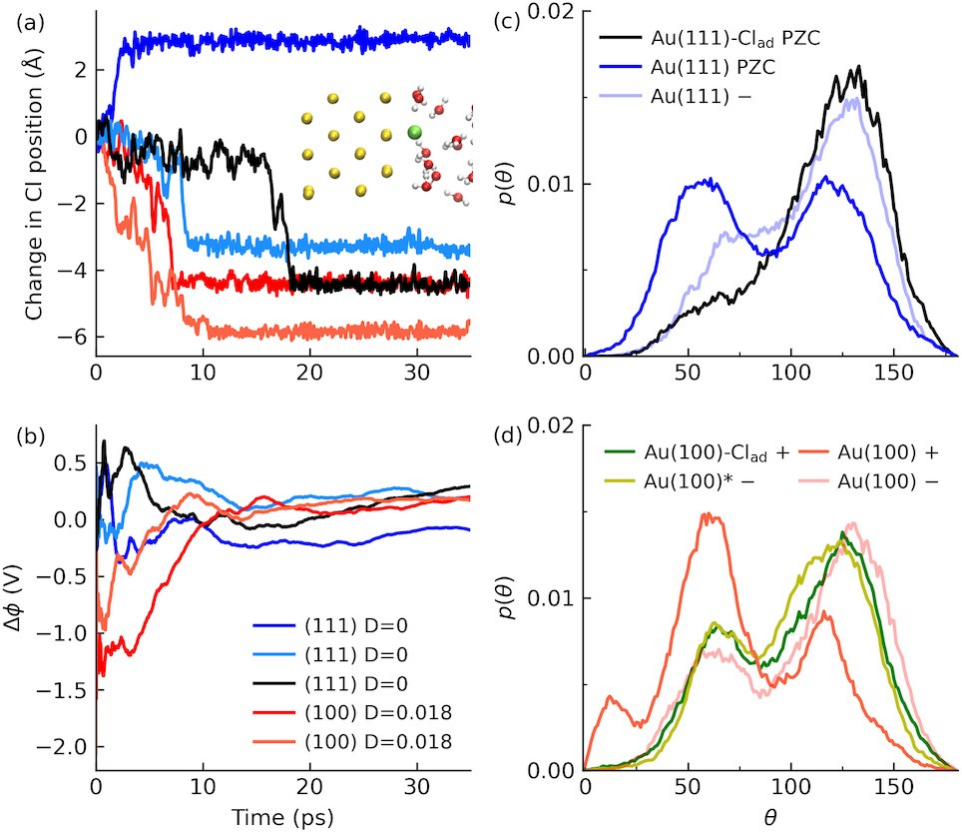
(a) Time evolution of Cl ions upon adsorption on both Au(111) at PZC (D=0 ) and Au(100) in finite‐field DFTMD simulations at D=0.018 au ($\sigma_{m}$ =8.2 *μ*C/cm^2^). Inset: a snapshot of the adsorbed Cl ion at gold electrode. (b) The corresponding evolution of the cell potential Δ*φ* (see Eq. 1 and text around for details). (c) Angular distributions between the water bisector and the surface normal for Au(111) with/without Cl adsorption at PZC (D=0 ). The corresponding distribution for cathodic polarized Au(111) at D=0.018 au is also shown. (d) Angular distributions between the water bisector and the surface normal for Au(100) with/without Cl adsorption at D=0.018 au. Results for cathodic polarized Au(100) are also shown and Au(100)* stands for the counter‐side of the gold slab with Cl adsorption.

We have also observed very rapid Cl adsorption on the Au(111) surface at PZC (D=0 ) and at polarized Au(100) (D=0.018 au). Figure 4a shows the change in position of the Cl ions over these DFTMD trajectories (independent ones from those presented in Figure 2). The Cl starts a few Å from the surface and adsorbs within a few ps and becomes stationary at the surface for the rest of the simulation. This comes in line with the “immeasurably high” adsorption rate deduced from the impedance spectroscopy.^[69]^ The observed fast Cl adsorption on the Au(111) surface at PZC instead of the Au(100) at PZC is likely due to a higher surface potential (therefore work function) of Au(111) as compared to that of Au(100). The opposite happened to polarized Au(111) and Au(100) surfaces are related to the interfacial water structure (see below).

The migration of Cl ions correlates well with the change in the potential difference across the simulation cell (Figure 4b). The movement of a negatively charged ion towards the surface results in a more positive potential from the increase in polarization. The correlation between the Cl migration towards the positive Au(100) surface and the increase in potential can be clearly seen from the corresponding trajectories. For one of the trajectories of Au(111) at D=0 , the Cl moves towards the other side of gold electrode, which leads to a more negative potential instead.

The change in the cell potential is also reflected in the water orientation at the interface. For Au(111) at PZC, the distribution of water orientation on the side of metal slab with Cl adsorption show a blue‐shift in the angle between water bisection and the surface normal and a clear diminishment in the peak around 60 degrees (Figure 4c). It further shows a similar distribution of water orientation at 130 degrees as observed in the cathodic polarized Au(111) surface at D=0.018 au but with enhanced intensity.

The situation is even more interesting in the case of polarized Au(100) with Cl adsorption. As shown in Figure 4d, distributions of water orientation at the cathodic polarized Au(100) and the anodic polarized Au(100) with Cl adsorption look almost the same. Given the size of cross‐section of our simulation cell, an integer charge of -1e will lead to a surface charge density of about −20 *μ*C/cm^2^. This means when one Cl ion adsorbed on an anodic polarized Au(100) with free charge of +8.2 *μ*C/cm^2^ (D=0.018 au), one would expect to see a charge inversion with almost the same amount but an opposite type of charge. This is exactly what we have seen for the case of Au(100) (Figure 4d). The corresponding oxygen density distributions confirming this point are shown in Figures S7 in the Supporting Information. The charge inversion with adsorbed Cl^−^ is expected to increase significantly the interfacial capacitance, as reported from the cyclic voltammetry.^[70]^ This signifies the ability of finite‐field DFTMD to simultaneously control the free charge via the electric displacement *D* and dynamically include the ionic charge due to surface chemistry. However, the amount of partial charge transfer can only be determined qualitatively (see below) and depend on the electric boundary conditions. It also does not escape our notice that this charge inversion due to specific adsorption of Cl^−^ at the anodic polarized Au(100) electrode provides an alternative explanation for the non‐monotonic change in the absorption THz spectra reported in recent experiments for gold electrode under positive bias^[6]^ and similar to that for chemisorbed OH on Pt(111).^[11]^

Furthermore, as mentioned before (Figure 2d), the 120 degrees peak of water orientation is clearly visible for anodic polarized Au(100) but completely disappears for anodic polarized Au(111). Upon the adsorption of Cl^−^, H‐bond network needs to response where the 130 degrees peak of water orientation shows up as a feature for both Au(111) and Au(100) (Figures 4c and d). This means the H‐bond networks on the anodic polarized Au(100) and the corresponding Cl‐adsorbed Au(100) look more similar than those on the anodic polarized Au(111) and the corresponding Cl‐adsorbed Au(111). Therefore, this structural feature of H‐bond network on the anodic polarized Au(100) is expected to facilitate the Cl adsorption as compared to the case of anodic polarized Au(111).

**Figure 5:**
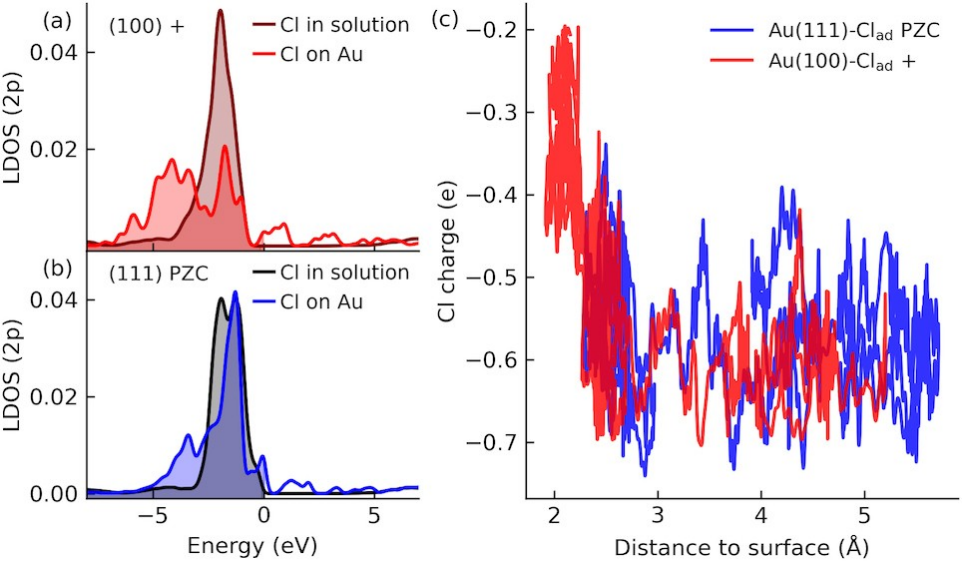
(a) and (b) The localized density of states (LDOS) for Cl in electrolyte solution and adsorbed on Au(100) and Au(111) respectively. The Fermi level is chosen to be zero. (c) Time evolution of atomic charge of Cl as a function of the distance to the Au electrode for both Au(100) and Au(111). The closest distance between Cl ion and Au electrode without specific adsorption is between 5.5 Å to 6.5 Å, see Figure S9 in the Supporting Information.

To gain further insights into the charge state of adsorbed Cl ions, we have also analyzed their electronic structures on the Au electrode and in the electrolyte solution. It is found that the 2*p* orbital of Cl is fully occupied when Cl^−^ is solvated in the electrolyte solution but it becomes split upon the adsorption at both Au(100) and Au(111) surfaces (Figures 5a and b). This indicates the nature of a strong covalent bond between Cl and Au electrode^[71]^ and the splitting is stronger for Au(100) than that for Au(111). These results by analyzing the density of states are further supported by the charge analysis using the restrained electrostatic potential (RESP) method for periodic systems.^[72]^ Despite that the atomic charge itself is not quite meaningful, the change in atomic charge does reflect the change in chemical bonding. It is found that the change in RESP charges before and after adsorption is more significant in the case of Au(100) as compared to Au(111) (Figure 5c). The percentage of partial charge transfer is about 50 % for adsorbed Cl^−^ on Au(100) and 20 % for that on Au(111), and the latter can be related to the experimentally measured electrosorption valency of 0.4e for Cl^−^ adsorption at gold electrode.[^73^, ^74^]

## Conclusion

We have demonstrated how the finite D‐field method can be used together with DFTMD simulations to describe electrified metal/electrolyte interfaces. This was done by simulating Au(100) and Au(111) electrodes in aqueous electrolyte of NaCl. The obtained Helmholtz capacitance for Au electrode of 60 *μ*F/cm^2^ agrees quite well with experimental results obtained from both impedance spectroscopy and cyclic voltammetry around PZC.

The high capacitance of Au without specific adsorbed ions is attributed to the electronic response of Au electrode by exploring the synergy in finite‐field simulations between DFT and semi‐classical Hamiltonians. It is found that semi‐classical simulations can reproduce the double‐layer capacitance obtained from DFTMD simulations by incorporating appropriate positions of the image plane. This does not only provide a physical account for the high capacitance often observed on the hydrophilic and metallic electrodes, but also point out strategies to fine‐tune force fields of ions in semi‐classical models for achieving a consistent structure–property relationship.

Unexpected fast Cl adsorptions were observed in the course of finite‐field DFTMD simulations. In particular, the adsorption of Cl ions on the anodic polarized Au(100) leads to a charge inversion, as shown by almost identical water orientational distributions on the anodic polarized Au(100) with Cl adsorption and the cathodic polarized Au(100) without Cl adsorption. This suggests a non‐monotonic change in the total charge for gold electrode under positive bias. The ability of finite‐field DFTMD to capture the distinction between free charge (from the metal electrode) via controlling the electric displacement *D* and ionic charge (from specifically adsorbed ions) via dynamical simulations opens doors to investigate their roles and couplings in surface reconstruction/dissolution, energy storage and catalytic processes.

## Supporting Information

Description of finite‐field methods, computational setups, relationship between image plane and Gaussian width parameter, density profiles, electrostatic potential profiles, RESP charge analysis and cumulative average of the cell potential.

## Conflict of Interests

The authors declare no conflict of interest.

1

## Supporting information

As a service to our authors and readers, this journal provides supporting information supplied by the authors. Such materials are peer reviewed and may be re‐organized for online delivery, but are not copy‐edited or typeset. Technical support issues arising from supporting information (other than missing files) should be addressed to the authors.

Supporting Information

## Data Availability

The data that supports the findings of this study are available in the supplementary material of this article.
