# Supplementary material for: Electronic Response and Charge Inversion at Polarized Gold Electrode
Source: Angew Chem Int Ed Engl. 2024 Nov 4;64(1):e202413614. doi: 10.1002/anie.202413614 (PMC11701363; doi:10.1002/anie.202413614)
Supplement: Supplementary file 1 — Supporting Information [file ANIE-64-e202413614-s001.pdf]

Angewandte Chemie
Eine Zeitschrift der Gesellschaft Deutscher Chemiker

Supporting Information

Electronic Response and Charge Inversion at Polarized Gold Electrode

*L. Andersson, M. Sprik, J. Hutter, C. Zhang**

Supporting Information: Electronic Response and Charge Inversion at Polarized Gold Electrode

Linnéa Andersson,[†] Michiel Sprik,[‡] Jürg Hutter,[¶] and Chao Zhang[*,†]

†*Department of Chemistry-Ångström Laboratory, Uppsala University, Lägerhyddsvägen 1, BOX 538, 75121 Uppsala, Sweden*

‡*Department of Chemistry, University of Cambridge, Lensfield Rd, Cambridge CB2 1EW, United Kingdom*

¶*Institut für Chemie, Universität Zürich, Winterthurerstrasse 190, CH-8057 Zürich, Switzerland*

E-mail: chao.zhang@kemi.uu.se

Finite-field methods

Finite displacement field coupling in DFTMD

Finite-field coupling for periodic systems was introduced by Stengel, Spaldin and Vanderbildt,[S1] based on the modern theory of polarization.[S2] In addition to the usual Kohn-Sham potential energy, a term which couples the D-field to the polarization is included,

$$U_{\mathrm{D}}[\gamma(\mathbf{r}),\mathbf{R}] = U_{\mathrm{PBC}}[\gamma(\mathbf{r}),\mathbf{R}] + \frac{\Omega}{8\pi}(D - 4\pi P_z[\gamma(\mathbf{r}),\mathbf{R}])^2, \tag{1}$$

where the polarization P_z depends on the nuclear positions $\mathbf{R} = \{\mathbf{R}_i\}$ and the wavefunction $\gamma(\mathbf{r})$. The polarization in a periodic crystal is multi-valued according to the modern theory of polarization,[S3] because the dipole moment depends on the choice of finite cell. Only the change in polarization, which can be measured from the current, is well-defined.

The electronic polarization is computed from the Berry phase[S4] using the momentum representation of the position operator $\mathbf{r} = i\frac{\partial}{\partial \mathbf{k}}$, which is equivalent to an integral over the Wannier centers:

$$\mathbf{P}_{el} = -\frac{2ei}{(2\pi)^3}\sum_n \int_{BZ} \langle u_{n\mathbf{k}}|\frac{\partial}{\partial \mathbf{k}}|u_{n\mathbf{k}}\rangle \, d\mathbf{k} = -\frac{2e}{\Omega}\sum_n \int w_n^*(\mathbf{r}) w_n(\mathbf{r})\mathbf{r} \, d\mathbf{r}. \tag{2}$$

The above representation requires evaluation over $\mathbf{k}$-points. In the limit of infinite cell size, $L_z \to \infty$, the above expression is equivalent to the Resta formula[S5] and the polarization along one direction can be calculated as:

$$P_{el} = -\frac{eL_z}{2\pi\Omega}\mathrm{Im}\ \ln\langle\gamma(\mathbf{r})|e^{i\frac{2\pi}{L_z}z}|\gamma(\mathbf{r})\rangle, \tag{3}$$

which is used in the CP2K implementation. Due to the lack of $\mathbf{k}$-point sampling, the polarization has a small dependence on the size of the simulation cell as shown in Figure S1. The CP2K implementations of finite-field DFTMD have been done previously[S6,S7] .

Finite displacement field coupling in semi-classical MD

The formation of the electric double layer requires long simulation times in the order of nanoseconds. For the relaxation of the metal-electrolyte interface, a semi-classical approach can be used which combines the speed of classical molecular dynamics (MD) with a polarizable electrode surface. In MetalWalls[S8] the electrode charges are described by Gaussian charge distributions of width $1/\sqrt{2}\eta$, while the electrolyte consists of point charges. The total charge density for a system with N electrolyte atoms with positions $\mathbf{r}_i$ and charges q_i and M electrode atoms with positions $\mathbf{r}_j$ and charges q_j is

$$\rho(\mathbf{r}) = \sum_{i=1}^{N} q_i \delta(\mathbf{r} - \mathbf{r}_i) + \sum_{j=1}^{M} q_j \eta^3 \pi^{3/2} \exp(-\eta^2 |\mathbf{r} - \mathbf{r}_j|^2). \tag{4}$$

The D-field coupled potential energy is

$$U_{\mathrm{D}}[\rho(\mathbf{r}), \mathbf{R}] = U_{\mathrm{PBC}}[\rho(\mathbf{r}), \mathbf{R}] + \frac{\Omega}{8\pi}(D - 4\pi P_z[\rho(\mathbf{r}), \mathbf{R}])^2 \tag{5}$$

where P_z can be calculated directly from the charge density since the charges are localized, and U_{PBC} consists of the Coulomb energy and the Lennard-Jones potential

$$U_{\mathrm{PBC}}[\rho(\mathbf{r}), \mathbf{R}] = \frac{1}{2} \int \frac{\rho(\mathbf{r})\rho(\mathbf{r}')}{|\mathbf{r} - \mathbf{r}'|} \, d\mathbf{r} + U_{LJ}(\mathbf{R}). \tag{6}$$

The potential energy of a metal(m)–electrolyte(e) system is a quadratic function of the electrode charges $\mathbf{Q} = [q_1, ..., q_M]$:

$$U_{\mathrm{D}}[\mathbf{Q}, \mathbf{R}] = U_{\text{m-m}} + U_{\text{m-e}} + U_{\text{e-e}} + \frac{\Omega}{8\pi}(D - 4\pi(P_{\mathrm{m}} + P_{\mathrm{e}}))^2 \tag{7}$$

$$= \frac{1}{2}\mathbf{Q}^\top \mathbf{A} \mathbf{Q} + \mathbf{Q}^\top \mathbf{b} + U_{\text{e-e}}(\mathbf{R}) + \frac{\Omega}{8\pi}(D - 4\pi(\mathbf{Q}^\top \mathbf{d} + P_e(\mathbf{R}))^2. \tag{8}$$

where $\mathbf{A}$ is the hardness kernel and $\mathbf{b}$ contains the integral contributions of $U_{\text{m-e}}$ as well as the vector to enforce the charge neutrality constraint.[S9,S10] The vector $\mathbf{d}$ is generated from

the integral to compute the dipole of Gaussian charges.

Therefore, in every time step the electrode charges can be optimized by solving a linear equation:

$$\frac{\partial U_{\mathrm{D}}[\mathbf{Q},\mathbf{R}]}{\partial \mathbf{Q}} = \mathbf{A}\mathbf{Q} + \mathbf{b} - \Omega\mathbf{d}(D - 4\pi(\mathbf{Q}^{\top}\mathbf{d} + P_e(\mathbf{R}))) = 0. \tag{9}$$

The implementation of D-field coupling in the MetalWalls code was done previously by the PHENIX group.[S11]

Computational setups

The dimensions of the Au(111) and Au(100) slabs are 3×4×4 (8.655 × 9.994 × 7.067 Å^3) and 3×3×4 (8.655 × 8.655 × 6.120 Å^3) respectively, with the experimental lattice constant of 4.08 Å. Slabs with a relatively small x-y plane area where chosen to sustain a large D-field during DFTMD. The values of the work functions with this setup come out quite reasonable, although that for the Au(111) is at bit overestimated (see SI Table S2). The electrolyte has an extent of 30 Å in the z-direction, consisting of 79 H_2O with 3 NaCl (2.1 mol/kg) for Au(111) and 71 H_2O with 3 NaCl (2.3 mol/kg) for Au(100). An initial electrolyte configuration was generated using Gromacs[S12] and the average mass density from DFTMD is shown in Figure S4 in the SI. The electrode/electrolyte systems were equilibrated with classical force fields (see Table S1) and MetalWalls[S8] using the default Gaussian width for Au ($1/\sqrt{2}\eta$=0.39 Å) over 10 ns at both PZC and under finite D field applied in the z-direction before performing DFTMD. Periodic boundary conditions were used in all directions. During the MetalWalls MD the gold slab was kept fixed in all directions and during DFTMD it was kept fixed in the z-direction. MetalWalls simulations with increased Gaussian width where performed with 30 layer gold slabs.

All DFT calculations and Born-Oppenheimer DFTMD simulations were performed with CP2K[S13] , where the electronic structure is described by Kohn-Sham density functional theory within the Gaussian and plane-wave framework[S14] . The core electrons are described by

Goedecker-Teter-Hutter GTH pseudopotentials[S15,S16] . The valence electrons are expressed by double-ζ basis sets with one set of polarization functions (DZVP) optimized for molecular systems.[S17] For the plane-wave expansion of the orbitals the energy cutoff was set to 500 Ry. The exchange-correlation functional is Perdew-Burke-Ernzerhof (PBE) including Grimme's dispersion corrections[S18] . All simulations were performed in the NVT ensemble with a target temperature of 330 K. The integration time step was 0.5 fs and the SCF (self-consistent field) convergence threshold was set to 10^{-6}. With finite-field coupling, diagonalization with Fermi-smearing cannot be used in the SCF optimization since the orbitals are not eigenfunctions of the Hamiltonian. Instead the orbital transformation (OT) method[S19] was used where the electronic structure is found by direct minimization of the energy functional under the constraint of orthonormal orbitals. This has been tested previously and applied successfully to extended metallic systems[S20] . The OT method was often used together with second generation Car-Parrinello (SGCP) MD to reduce the computational cost for metallic systems.[S21] However, in the case of finite-field methods, a tight SCF convergence is required for polarization, which is why regular BOMD was used instead.

Table S1: Lennard-Jones parameters and charges used in the MetalWalls simulations. The Lorentz-Berthelot rule was used for mixed interactions. For water the SPC/E model[S22] was used with an OH-bond constraint of 1 Å and and HOH angular constraint of 109.47 °.

Parameter	O	H	Na	Cl	Au
Charge (e)	-0.8476	0.4238	1	-1	0
ϵ (kJ/mol)	0.6502	0	0.4184	0.4184	22.13336
σ (Å)	3.166	0	2.584	4.401	2.951

Work function for different slab dimensions

Table S2: Calculated work functions for different dimensions of the Au slabs.

Crystal face	Dimensions	Work function (eV)
111	3×4×4	5.84
111	3×4×6	5.27
100	3×3×4	5.35
100	3×3×6	5.76
100	4×4×4	4.87

Cell size dependence of the polarization

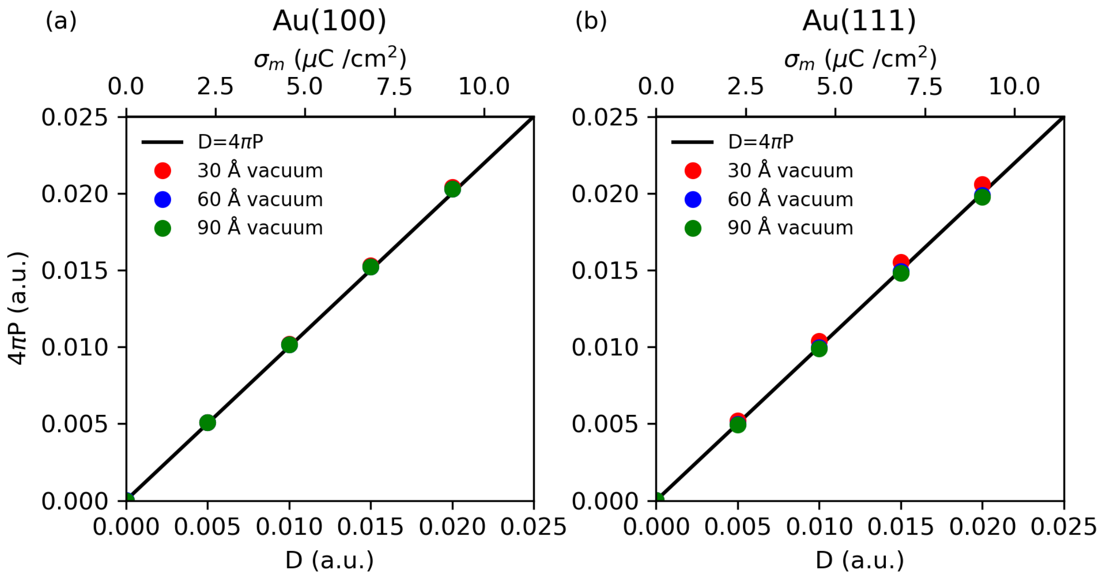

Figure S1: Scaling of the polarization of the Au(100) 3×3×4 and Au(111) 3×4×4 slabs with the D-field intensity calculated with DFT in CP2K for different amounts of vacuum in the periodic cell.

Surface charge density in DFT and semi-classical Hamiltonians

Table S3: The surface charge density for the Au slabs in 90 Å vacuum under the field strength D=0.018 a.u. . The theoretical charge density is given by $\sigma_m = D/4\pi$ (8.20 μC/cm^2).

Crystal face	Level of theory	Gaussian width $(\sqrt{2}\eta)^{-1}$ (Å)	Charge density (μC/cm^2)
100	DFT	-	8.32
100	Semi-classical	0.39	8.20
100	Semi-classical	1.20	8.20
111	DFT	-	8.06
111	Semi-classical	0.39	8.20
111	Semi-classical	1.48	8.20

Relationship between image plane and Gaussian width parameter

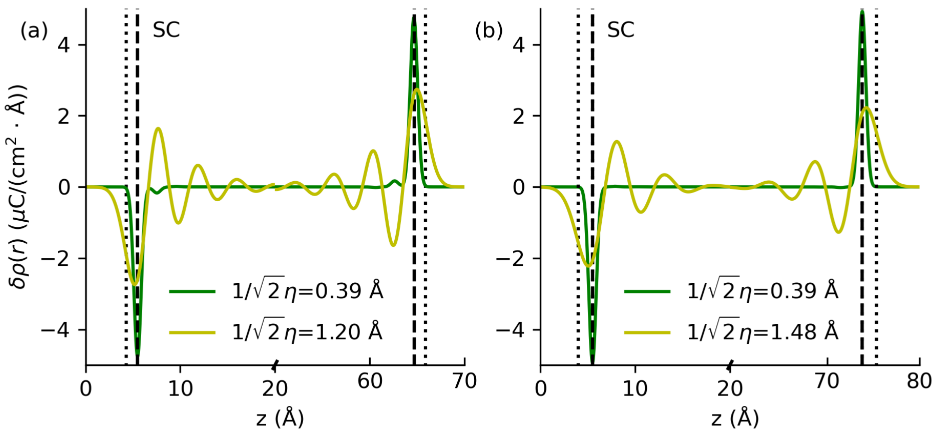

Figure S2: Charge response from the semi-classical (SC) model with different Gaussian widths for both Au(100) and Au(111) slabs in vacuum at $D = 0.018$ a.u. . Atomic plane: dashed line; image plane: dotted line. For $1/\sqrt{2}\eta = 0.39$ Å, the atomic plane z_a and the image plane z_{im} coincide with each other and the charge response is symmetric. With an increased Gaussian width, the charge distributions on different atomic planes start to overlap and the charge response on the gold surface becomes asymmetric, similar to the DFT results.

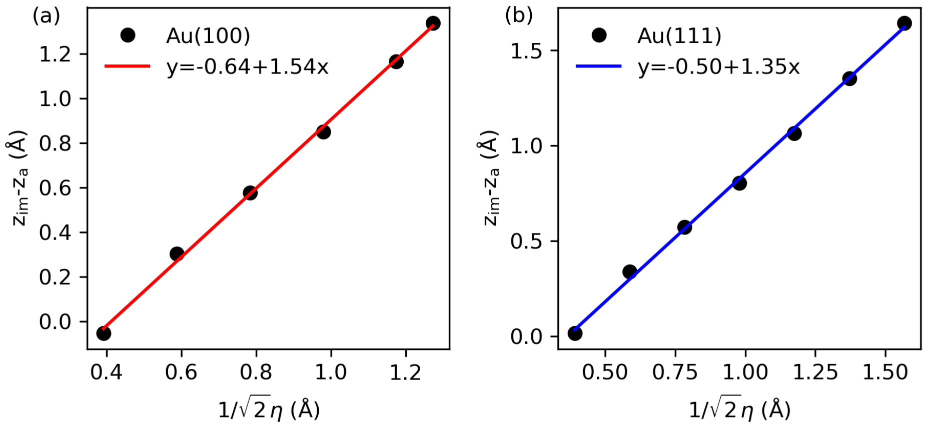

Figure S3: Relationship between the Gaussian width $1/\sqrt{2}\eta$ and the distance between the image plane and the atomic plane in MetalWalls for both Au(100) and Au(111) surfaces.

Density profiles

Total density profile

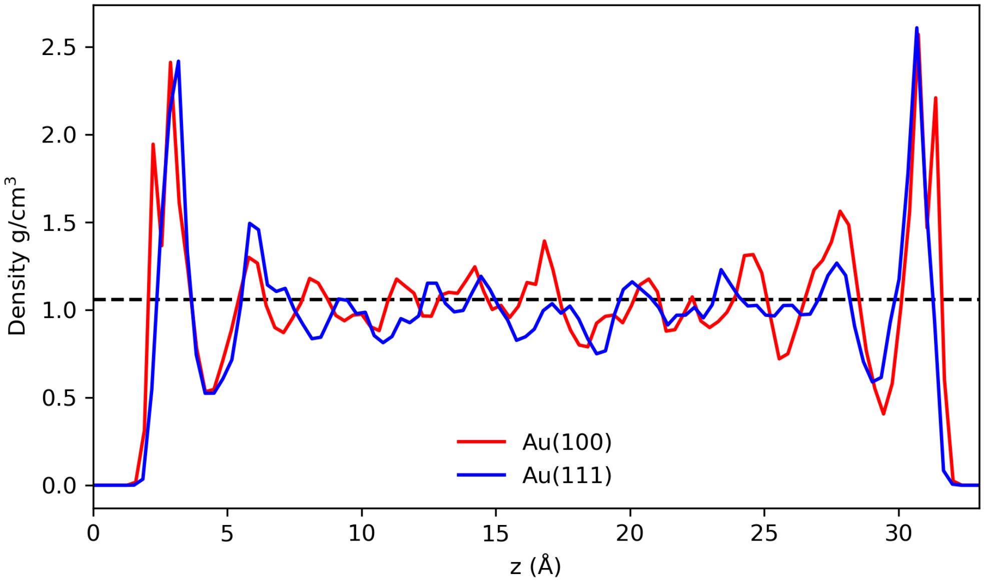

Figure S4: Mass density from DFTMD for Au(100) and Au(111) at D=0 a.u., averaged over 3 trajectories. The dotted line shows the experimental density of a 2 mol/kg NaCl(aq) solution.[S23]

Number density profiles

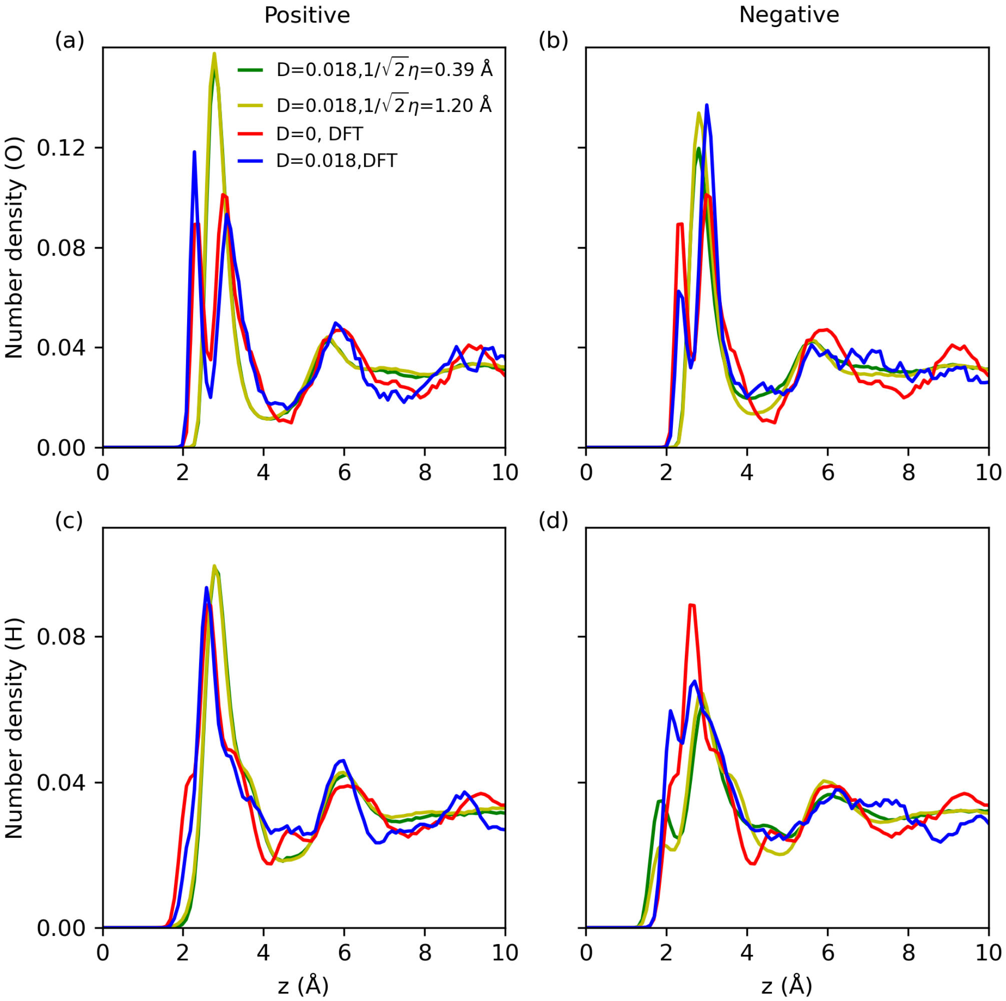

Figure S5: Distributions of oxygen and hydrogen at the Au(100) surface. For the simulations at D=0.018 a.u., panels (a)-(c) show the positive side distributions and (b)-(d) show the negative side distributions.

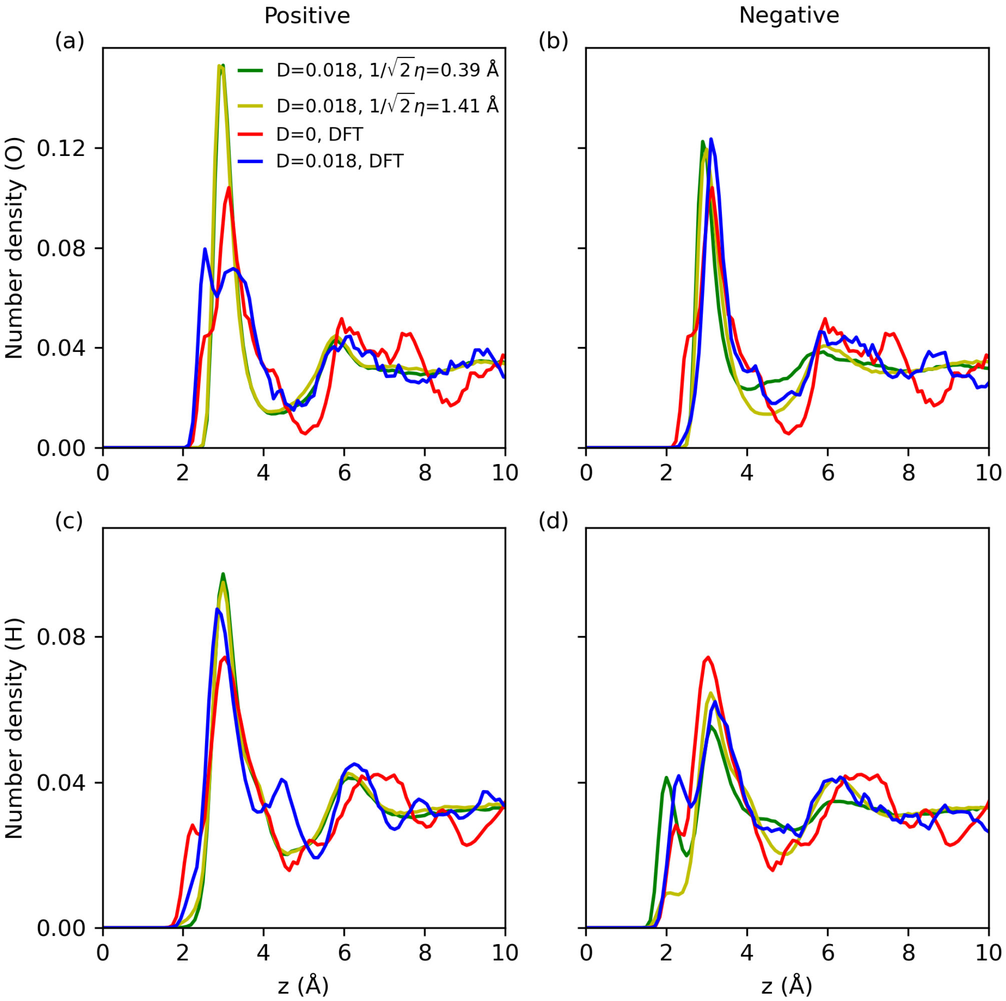

Figure S6: Distributions of oxygen and hydrogen at the Au(111) surface. For the simulations at D=0.018 a.u., panels (a)-(c) show the positive side distributions and (b)-(d) show the negative side distributions. The D=0 distribution comes from a simulation of Au(111)/water system to eliminate any specific ion adsorption.

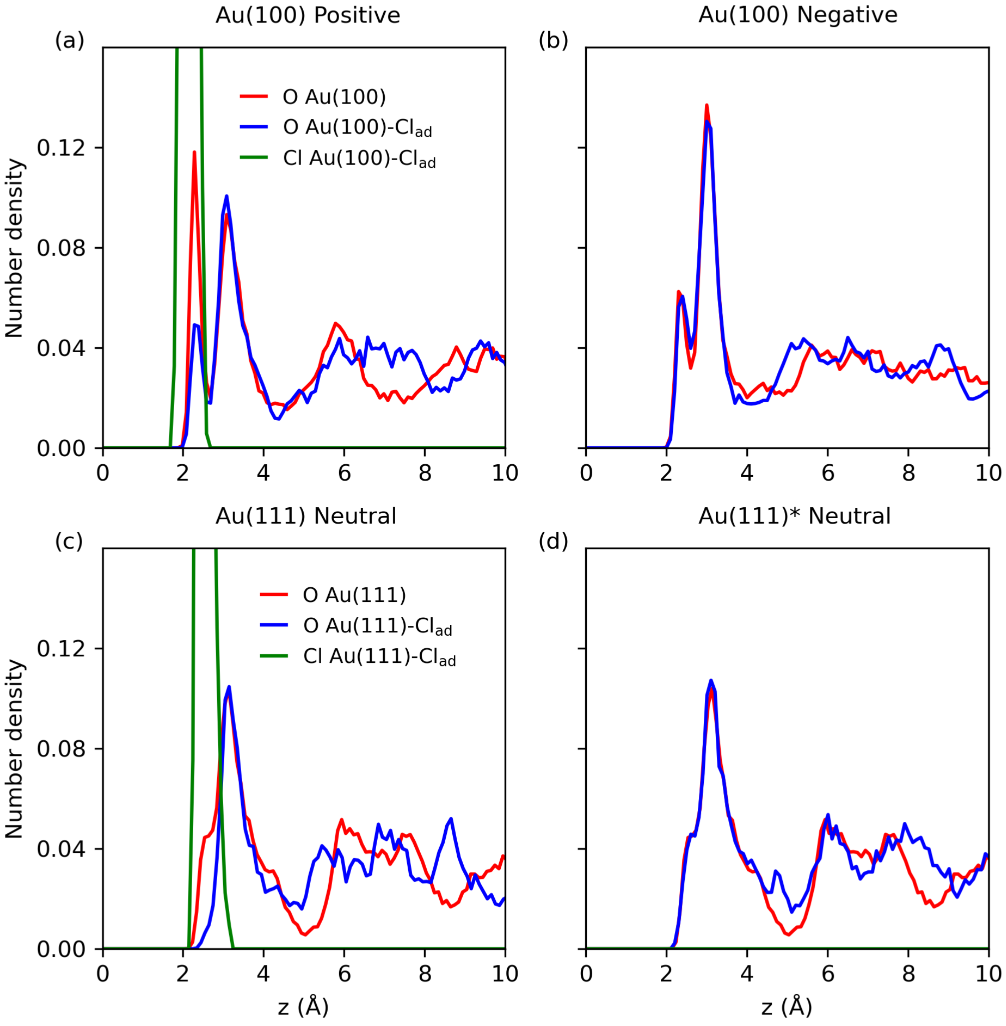

Figure S7: Oxygen and Cl distributions for the Au(100)-Cl$_{ad}$ at $D = 0.018$ a.u. (a) and Au(111)-Cl$_{ad}$ surfaces at $D = 0$ a.u. (c). The distributions at the opposite surface for each system (i.e. Au(100) Negative and Au(111)* Neutral) are also shown in (b)-(d).

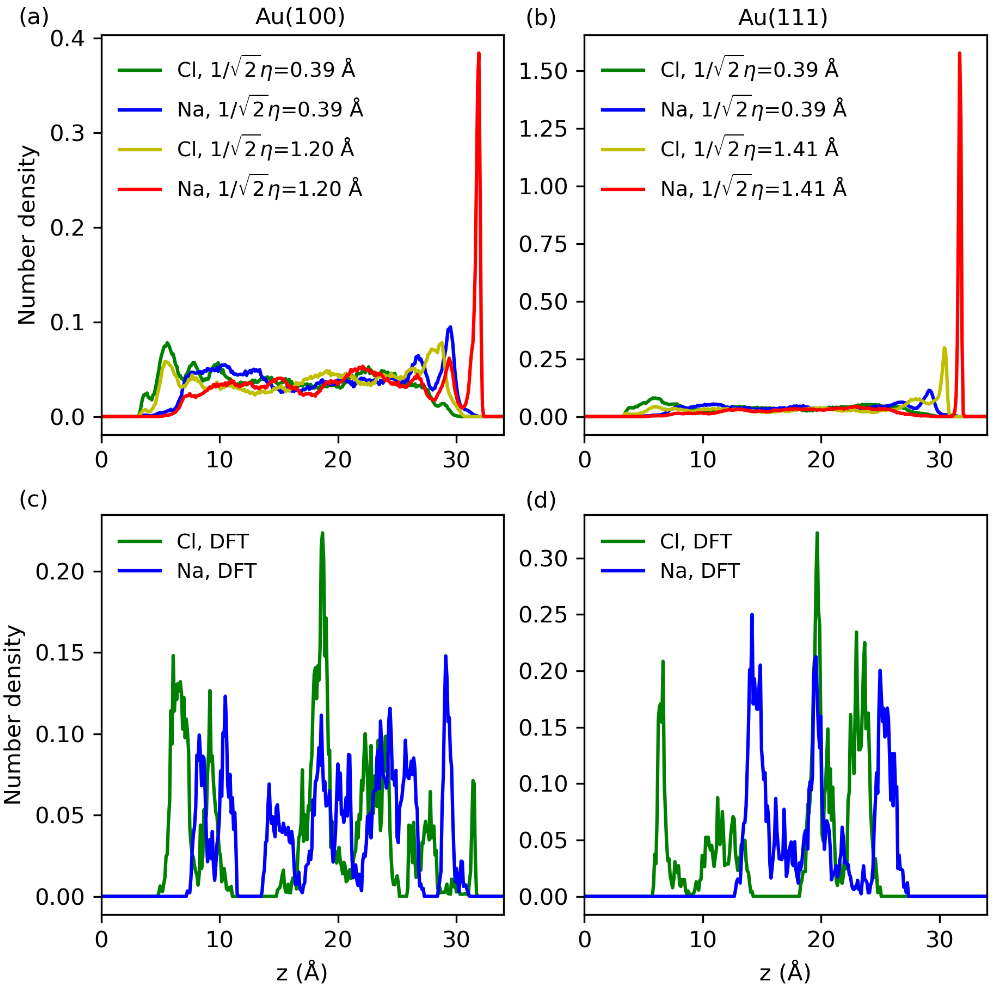

Figure S8: Distributions of Na and Cl at D=0.018 from semi-classical simulations (a)-(b) and from DFTMD (c)-(d). The positive Au surface is located at 0 Å and the negative surface at 34 Å.

Counter-ion positions

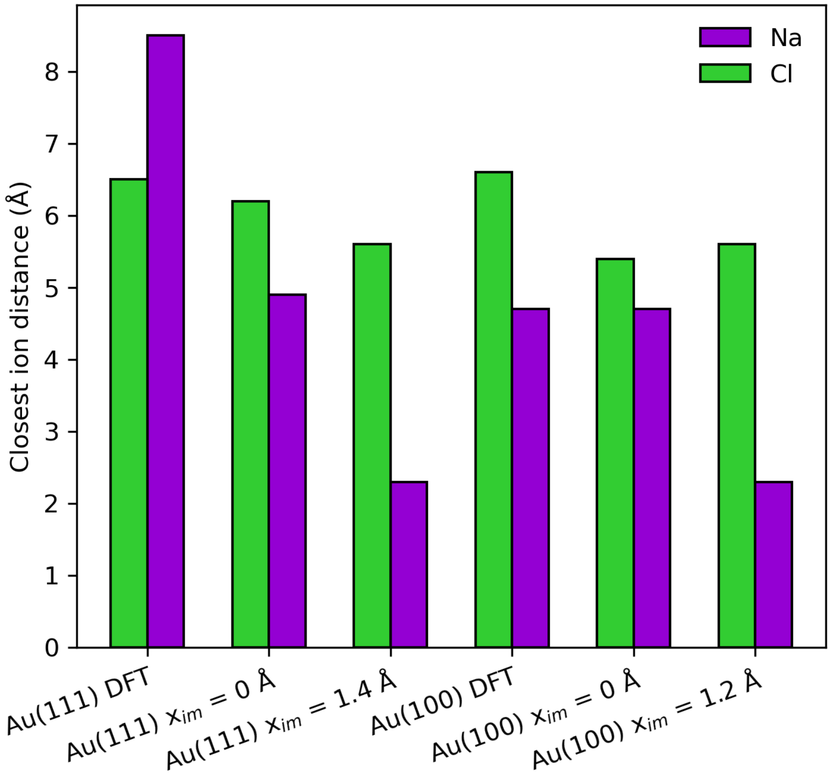

Figure S9: Average distance between the first number density peak of counter-ion and charged surfaces from both finite-field DFTMD and finite-field semi-classical MD. For the positively charged surfaces, only the positions of Cl are shown; For the negatively charged surfaces, only the positions of Na are shown. DFTMD results refer to the systems without specific ion adsorption, i.e. Figure 1 in the Main Text.

Electrostatic Potential profiles

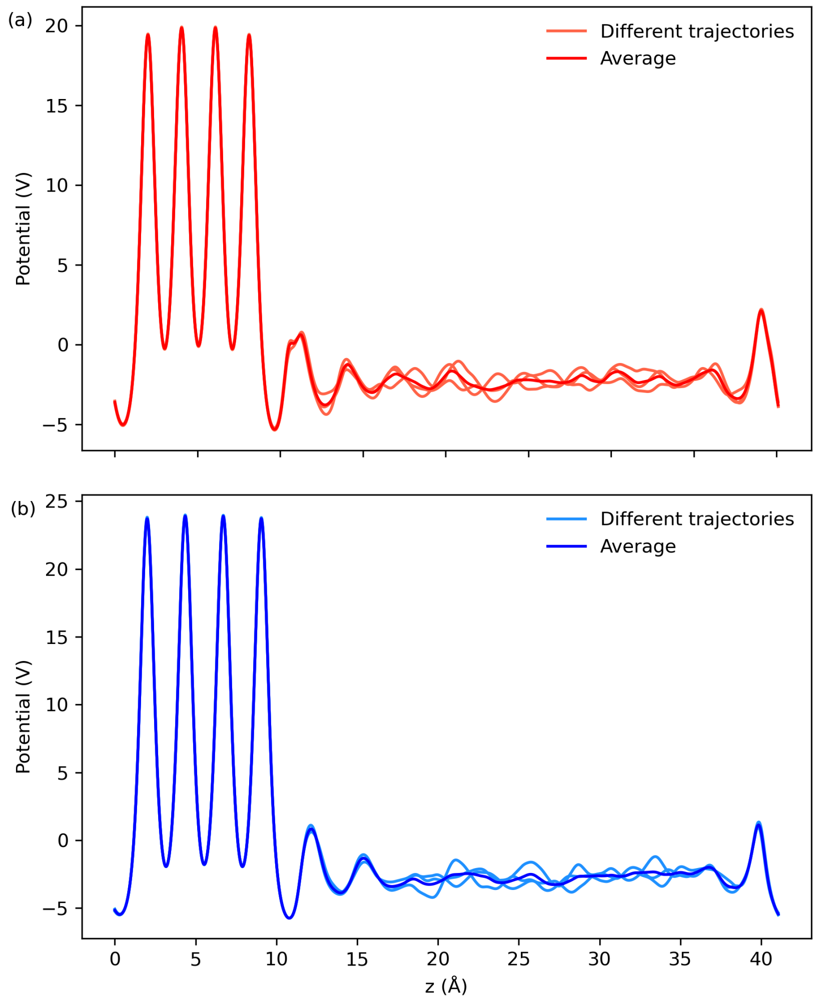

Figure S10: Electrostatic Potential profiles from DFTMD simulations of Au(100) (a) and Au(111) (b) at D=0.018 a.u. .

Cumulative average of the cell potential $\Delta\phi$

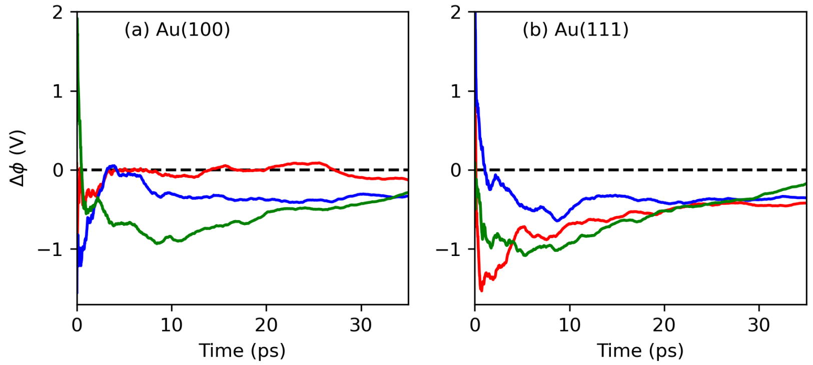

Figure S11: Cumulative average of the cell potential $\Delta\phi$for each individual trajectory in the cases of Au(100) in panel a) and Au(111) in panel b). The cumulative average over three trajectories in each case was shown in Figure 2c of the Main Text.

RESP charge analysis

Table S4: Average RESP charges of Cl in solution and adsorbed on Au. $q_{\mathrm{A}u}^{\mathrm{tot}}$ is the total charge of the Au slab with and without Cl adsorbed.

System	q_{Cl} (sol)	q_{Cl} (Au)	$q_{\mathrm{A}u}^{\mathrm{tot}}$	$q_{\mathrm{A}u}^{\mathrm{tot}}$ (Cl)
Au(100) D=0.018	-0.6	-0.3	0.12	0.08
Au(111) D=0	-0.6	-0.5	0.55	0.48

References

(S1) Stengel, M.; Spaldin, N. A.; Vanderbilt, D. Electric displacement as the fundamental variable in electronic-structure calculations. *Nat. Phys.* **2009**, *5*, 304–308.

(S2) King-Smith, R. D.; Vanderbilt, D. Theory of polarization of crystalline solids. *Phys. Rev. B* **1993**, *47*, 1651.

(S3) Spaldin, N. A. A beginner's guide to the modern theory of polarization. *J. Solid State Chem.* **2012**, *195*, 2–10.

(S4) Resta, R. Macroscopic polarization in crystalline dielectrics: the geometric phase approach. *Rev. Mod. Phys.* **1994**, *66*, 899.

(S5) Resta, R. Quantum-Mechanical Position Operator in Extended Systems. *Phys. Rev. Lett.* **1998**, *80*, 1800.

(S6) Zhang, C.; Hutter, J.; Sprik, M. Computing the Kirkwood g-Factor by Combining Constant Maxwell Electric Field and Electric Displacement Simulations: Application to the Dielectric Constant of Liquid Water. *J. Phys. Chem. Lett.* **2016**, *7*, 2696–2701.

(S7) Zhang, C.; Hutter, J.; Sprik, M. Coupling of Surface Chemistry and Electric Double Layer at TiO_2 Electrochemical Interfaces. *J. Phys. Chem. Lett.* **2019**, *10*, 3871–3876.

(S8) Marin-Laflèche, A.; Haefele, M.; Scalfi, L.; Coretti, A.; Dufils, T.; Jeanmairet, G.; Reed, S. K.; Serva, A.; Berthin, R.; Bacon, C.; Bonella, S.; Rotenberg, B.; Madden, P. A.; Salanne, M. MetalWalls: A classical molecular dynamics software dedicated to the simulation of electrochemical systems. *J. Open Source Softw.* **2020**, *5*, 2373.

(S9) Scalfi, L.; Salanne, M.; Rotenberg, B. Molecular Simulation of Electrode-Solution Interfaces. *Annu. Rev. Phys. Chem.* **2021**, *72*, 189 – 212.

(S10) Shao, Y.; Andersson, L.; Knijff, L.; Zhang, C. Finite-field coupling via learning the charge response kernel. *Electron. Struc.* **2022**, *4*, 014012.

(S11) Dufils, T.; Sprik, M.; Salanne, M. Computational Amperometry of Nanoscale Capacitors in Molecular Simulations. *J. Phys. Chem. Lett.* **2021**, *12*, 4357 – 4361.

(S12) Van Der Spoel, D.; Lindahl, E.; Hess, B.; Groenhof, G.; Mark, A. E.; Berendsen, H. J. GROMACS: Fast, flexible, and free. *J. Comput. Chem.* **2005**, *26*, 1701–1718.

(S13) Kühne, T. D.; Iannuzzi, M.; Del Ben, M.; Rybkin, V. V.; Seewald, P.; Stein, F.; Laino, T.; Khaliullin, R. Z.; Schütt, O.; Schiffmann, F.; Golze, D.; Wilhelm, J.; Chulkov, S.; Bani-Hashemian, M. H.; Weber, V.; Borštnik, U.; Taillefumier, M.; Jakobovits, A. S.; Lazzaro, A.; Pabst, H.; Müller, T.; Schade, R.; Guidon, M.; Andermatt, S.; Holmberg, N.; Schenter, G. K.; Hehn, A.; Bussy, A.; Belleflamme, F.; Tabacchi, G.; Glöß, A.; Lass, M.; Bethune, I.; Mundy, C. J.; Plessl, C.; Watkins, M.; VandeVondele, J.; Krack, M.; Hutter, J. CP2K: An electronic structure and molecular dynamics software package -Quickstep: Efficient and accurate electronic structure calculations. *J. Chem. Phys.* **2020**, *152*.

(S14) Vandevondele, J.; Krack, M.; Mohamed, F.; Parrinello, M.; Chassaing, T.; Hutter, J. QUICKSTEP: Fast and accurate density functional calculations using a mixed Gaussian and plane waves approach. *Comput. Phys. Commun.* **2005**, *167*, 103–128.

(S15) Goedecker, S.; Teter, M. Separable dual-space Gaussian pseudopotentials. *Phys. Rev. B* **1996**, *54*, 1703.

(S16) Hartwigsen, C.; Goedecker, S.; Hutter, J. Relativistic separable dual-space Gaussian pseudopotentials from H to Rn. *Phys. Rev. B* **1998**, *58*, 3641.

(S17) VandeVondele, J.; Hutter, J. Gaussian basis sets for accurate calculations on molecular systems in gas and condensed phases. *J. Chem. Phys.* **2007**, *127*, 114105.

(S18) Goerigk, L.; Grimme, S. A thorough benchmark of density functional methods for general main group thermochemistry, kinetics, and noncovalent interactions. *Phys. Chem. Chem. Phys.* **2011**, *13*, 6670–6688.

(S19) VandeVondele, J.; Hutter, J. An efficient orbital transformation method for electronic structure calculations. *J. Chem. Phys.* **2003**, *118*, 4365–4369.

(S20) Santarossa, G.; Vargas, A.; Iannuzzi, M.; Pignedoli, C. A.; Passerone, D.; Baiker, A. Modeling bulk and surface Pt using the "Gaussian and plane wave" density functional

theory formalism: Validation and comparison to k-point plane wave calculations. *J. Chem. Phys.* **2008**, *129*, 234703.

(S21) Musso, T.; Caravati, S.; Hutter, J.; Iannuzzi, M. Second generation Car-Parrinello MD: application to the h-BN/Rh(111) nanomesh. *Eur. Phys. J. B* **2018**, *91*, 148.

(S22) Berendsen, H. J. C.; Grigera, J. R.; Straatsma, T. P. The missing term in effective pair potentials. *J. Phys. Chem.* **1987**, *91*, 6269–6271.

(S23) Rumble, J. *CRC Handbook of Chemistry and Physics*; CRC Handbook of Chemistry and Physics; CRC Press: Boca Raton, FL, 2018.
